# High levels of EPHB2 expression predict a poor prognosis and promote tumor progression in endometrial cancer

**DOI:** 10.1515/biol-2025-1172

**Published:** 2025-11-20

**Authors:** Yanlai Xiao, Jian Wang, Xiangzhai Zhao, Jie Xu, Huan Zhao, Zhaojun Guo, Jun Zhao, Yajing Zhang, Ruoxi Wang, Yiwei Zhang

**Affiliations:** Department of Obstetrics and Gynecology, Hebei Medical University Third Hospital, Shijiazhuang, 050051, Hebei, China; Department of Obstetrics and Gynecology, Hebei Medical University Third Hospital, 139 Ziqiang Road, Shijiazhuang, 050051, Hebei, China

**Keywords:** endometrial cancer, tumor progression, prognosis, immune infiltration, EPHB2

## Abstract

Endometrial cancer (EC) exhibits increasing incidence and mortality, necessitating novel prognostic biomarkers and therapeutic targets. This study systematically investigates EPHB2 as a potential biomarker through comprehensive bioinformatics (TIMER 2.0, Human Protein Atlas, Xanadu Academic Online, Sento Academic Online, TCGA, GeneMANIA, GSEA, BEST database, and SCAR database) and experimental analyses (si-EPHB2 and OE-EPHB2 RL95-2 cell models with RT-qPCR, western blot, CCK-8, wound healing, Transwell, and TUNEL assays). Our findings demonstrate that EPHB2 is significantly overexpressed in EC, correlating with advanced pathological grade, histological type, and poor prognosis, while its high expression activates PI3K/AKT/MAPK signaling and promotes proliferation, migration, invasion, and suppresses apoptosis; conversely, EPHB2 knockdown exhibits opposite effects, revealing its critical role in EC progression through immune modulation and oncogenic signaling activation, thereby establishing EPHB2 as a promising therapeutic target for EC treatment.

## 1 Introduction

Endometrial cancer (EC) is the most common gynecological malignancy worldwide, predominantly affecting postmenopausal women [1,2]. Its incidence is increasing, with approximately 417,000 new cases and 97,000 deaths reported globally in 2020 [1,3,4]. Incidence rates are highest in North America, Europe, and Oceania, influenced by risk factors such as obesity, low parity, and genetic predispositions like Lynch syndrome. The growing burden of EC underscores the need for increased awareness, early detection, and improved treatment strategies [5–7]. Personalized therapy and biomarkers play a crucial role in enhancing the management of EC [8]. Tailoring treatment regimens to an individual’s genetic, molecular, and clinical characteristics can increase efficacy, reduce side effects, and improve patient prognosis [9,10]. However, challenges such as the limited availability and validity of biomarkers remain.

Biomarkers play a crucial role in the diagnosis, prognostic assessment, therapeutic decision-making, and monitoring of disease progression in EC [11,12]. For example, gene mutation markers such as p53 [13] and PTEN [14] can predict prognosis, while estrogen and progesterone receptors guide treatment selection [15]. However, many biomarkers are expressed across different cancer types and lack specificity, leading to misdiagnosis or underdiagnosis. Additionally, biomarkers often fail to detect low levels of tumor cells accurately, particularly in early detection.

Diagnostic accuracy can improve when using multiple markers together. For example, combining molecular markers (like gene mutations) with protein markers (like receptor expression) increases specificity and sensitivity [16,17]. EC biomarkers show high heterogeneity. Marker expression varies greatly between patients. It can also differ within the same patient. Differences may occur at different tumor sites or time points [18,19]. It is worth noting that translating biomarkers from research into clinical applications faces many challenges. These include designing robust clinical trials, obtaining regulatory approvals, and managing economic costs. Therefore, the discovery and validation of more efficient biomarkers is particularly urgent.

Ephrin type-B receptor 2 (EPHB2) is a receptor tyrosine kinase in the EPH family, playing a crucial role in cell-to-cell communication, cell morphology, motility, and localization [20]. EPHB2 influences cell behavior by triggering intracellular signaling pathways through binding to its ligand, ephrin-B. Studies on the expression and function of EPHB2 in various tumors have demonstrated its dual role in tumorigenesis and progression, acting as both a tumor suppressor and an oncogene, depending on the tumor type and microenvironment [20]. For example, in colorectal cancer, EPHB2 often exhibits a tumor-suppressive role, and its inactivation correlates with tumor progression and invasion; whereas in prostate cancer, EPHB2 may promote tumor growth and metastasis [21–23]. However, the role and function of EPHB2 in EC remain unclear. Therefore, studying the role of EPHB2 in EC not only helps improve prognostic prediction accuracy but also provides a scientific basis for developing novel therapeutic strategies, which holds significant clinical and research value.

In summary, this study aims to elucidate the significance and function of EPHB2 in EC using bioinformatics and experimental validation. The findings are expected to establish a theoretical research foundation supporting EPHB2 as a potential biomarker for predicting prognosis and guiding treatment strategies in EC.

## 2 Materials and methods

### 2.1 Expression analysis of EPHB2

We utilized multiple databases to perform a comprehensive analysis of EPHB2 expression. The TIMER 2.0 database [24] was employed to examine the pan-cancer expression of EPHB2. To determine the cellular localization of EPHB2 and its protein expression in EC tissues versus normal tissues, we used the Human Protein Atlas database (https://www.proteinatlas.org/). Additionally, the Sento Academic Online tool (https://www.xiantaozi.com/) was used to assess the mRNA expression levels of EPHB2 in EC and normal tissues (these databases aggregate extensive datasets from the TCGA and GTEx projects, facilitating more sophisticated and integrative analyses).

### 2.2 Analyzing the clinical relevance of EPHB2 in EC

In this study, we utilized the Sento Academic Online tool to investigate the relationship between EPHB2 expression and various clinical parameters in EC. Specifically, we examined the correlation between EPHB2 expression and pathological grade, pathological type, and clinical stage using data derived from the TCGA-UCEC cohort (*n* = 589: 560 tumor samples and 29 adjacent normal tissues) and GTEx normal tissue samples.

### 2.3 Prognostic analysis and modeling of EPHB2 in EC

In this study, we utilized the Sento Academic Online Tool to assess the correlation between EPHB2 expression in EC and patient outcomes, including overall survival (OS), disease-specific survival (DSS), and progression-free interval (PFI) risk classification, based on data from the TCGA and GTEx projects. Furthermore, we developed a prognostic model using EPHB2 gene expression as a Cox proportional hazards factor, leveraging EC data from the TCGA through the Sento Academic Online Tool. The model’s accuracy was subsequently validated using a prognostic calibration curve.

### 2.4 EPHB2 protein–protein interaction (PPI) network construction and GSEA functional enrichment in EC

We employed GeneMANIA [25] to identify proteins interacting with EPHB2 and to construct the corresponding PPI network. Additionally, we conducted functional enrichment analyses using the GSEA feature of the BEST online database [26]. Specifically, we performed Gene Ontology (GO), Kyoto Encyclopedia of Genes and Genomes (KEGG), and Hallmark pathway enrichment analyses on the top 500 genes associated with EPHB2.

### 2.5 Genomic mutation analysis of EPHB2 in EC

This study utilized the genomic mutation analysis feature of the BEST online database to investigate mutations in the EPHB2 gene in EC.

### 2.6 Analysis of EPHB2 immune infiltration in EC

Using the Sento Academic Online tool, we assessed the immune infiltration landscape in EC by analyzing the percentage of various immune cell types in patients with high and low EPHB2 expression. Additionally, the correlation between EPHB2 expression and immune cell infiltration was determined using the single-sample gene set enrichment analysis algorithm.

### 2.7 Single-cell analysis of EPHB2 in EC

In this study, we utilized the SCAR (Cancer Database for the Single Cell Transcriptome) [27] to determine and visualize the expression pattern of EPHB2 in a single-cell dataset of EC.

### 2.8 Cell culture

Normal endometrial epithelial cells (HESC) and various EC cell lines (Ishikawa, ECC-1, Hec-50B, KLE, and RL95-2) were obtained from the Shanghai Cell Bank, Chinese Academy of Sciences. These cells were cultured in DMEM, F12K, or 1640 complete medium, each supplemented with 10% fetal bovine serum (FBS) and 1% penicillin–streptomycin. Cultures were maintained at 37°C in a 5% CO_2_ incubator, with regular medium changes to ensure optimal cell health and growth.

### 2.9 Cell transfection

We established an RL95-2 cell line with silenced EPHB2 expression (si-EPHB2). GenePharma (Shanghai, China) synthesized three pairs of small interfering RNAs (siRNAs) targeting EPHB2: si-EPHB2-1 (sense: 5′-GAAGAAACGCUAAUGGACUUUU-3′, antisense: 5′-AGUCCAUUAGCGUUCUU-3′), si-EPHB2-2 (sense: 5′-GAUGAGAACAUGAACACGAUU-3′, antisense: 5′-UCGUGUUCAUGUUCUCAUCUU-3′), and si-EPHB2-3 (sense: 5′-GUGCAACGUGUUUGAGUCAUU-3′), antisense: 5′-UGACUCAAACACGUUGCACUU-3′). Transfection was conducted using Lipofectamine™ 3000 (Invitrogen; Cat. No. L3000015) following the manufacturer’s protocol. RL95-2 cells were seeded in six-well plates at a density of 2 × 10^5^ cells/well and cultured overnight until reaching 70–80% confluency. Each well received a mixture of 5 µL Lipofectamine™ 3000 reagent diluted in 125 µL Opti-MEM™ I Reduced Serum Medium (Gibco; Cat. No. 31985-070), incubated for 5 min at room temperature, and 50 pmol of each si-EPHB2 RNA diluted in 125 µL Opti-MEM™ I medium. After a 20 min incubation to form the siRNA–lipid complex, the mixture was added dropwise to cells in each well, gently mixed, and incubated for 6 h at 37°C in a 5% CO_2_ incubator. Subsequently, the medium was replaced with fresh complete medium. For overexpression, DMEM without antibiotics was used instead of the growth medium, and then the EPHB2 overexpression plasmid and its corresponding empty vector control (OE-NC) were transfected into RL95-2 cells using Lipofectamine™ 3000.

### 2.10 RT-qPCR

Total cellular RNA was extracted using the TRIzol™ Reagent kit (Thermo Fisher Scientific, USA; Cat. No. 15596018CN). RNA purity and concentration were measured with a spectrophotometer, and concentrations were adjusted with diethyl pyrocarbonate-treated water (Thermo Fisher Scientific, USA; Cat. No. AM9915G). The RNA was reverse transcribed into cDNA using the PrimeScript™ RT reagent Kit (Takara, Japan; Cat. No. RR037A). EPHB2 expression was analyzed using GAPDH as the internal control, with primers synthesized by Sangon Biotech (Shanghai) Co., Ltd, China. RT-qPCR was performed with SYBR^®^ Premix Ex Taq™ II (Takara, Japan; Cat. No. RR820A) under the following conditions: 95°C for 10 min, followed by 40 cycles of 95°C for 15 s and 60°C for 1 min. Each sample was tested in triplicate. EPHB2 Primers: Forward: 5′-CCACTCATCATCGGCTCCTC-3′, Reverse: 5′-GCTCAAACCCCCGTCTGTTA-3′; GAPDH Primers: Forward: 5′-ACAACTTTGGTATCGTGGAAGG-3′, Reverse: 5′-GCCATCACGCCACAGTTTC-3′.

### 2.11 Western blot analysis

Total protein was extracted using RIPA lysis buffer (Thermo Fisher Scientific, USA; Cat. No. 89900) with a protease and phosphatase inhibitor cocktail (Thermo Fisher Scientific, USA; Cat. No. 78440). Protein concentrations were measured with the BCA Protein Assay Kit (Thermo Fisher Scientific, USA; Cat. No. 23227). Proteins were separated by SDS-PAGE and transferred to PVDF membranes (Millipore, USA; Cat. No. IPVH00010). Membranes were blocked with 5% non-fat milk in TBST for 1 h, then incubated overnight at 4°C with primary antibodies: EPHB2 (Abcam; Cat. No. ab124924), PI3K (Cell Signaling Technology; Cat. No. 4249), p-PI3K (Cell Signaling Technology; Cat. No. 17366), AKT (Cell Signaling Technology; Cat. No. 9272), p-AKT (Cell Signaling Technology; Cat. No. 4060), MAPK (Cell Signaling Technology; Cat. No. 9102), p-MAPK (Cell Signaling Technology; Cat. No. 4370), and GAPDH (Cell Signaling Technology; Cat. No. 5174). After washing, membranes were incubated with HRP-conjugated secondary antibodies (Cell Signaling Technology, USA) for 1 h. Protein bands were visualized using an ECL detection system (Thermo Fisher Scientific, USA; Cat. No. 32106) and quantified with ImageJ software.

### 2.12 CCK-8 assay

RL95-2 EC cells transfected were seeded into 96-well plates at a density of 5,000 cells per well. After 24 h of incubation, cell viability was assessed by measuring the absorbance at 460 nm using the CCK-8 Assay Kit (Sunn Bio, Wuhan, China; Cat. No. SNK-006). This experiment was performed to evaluate the impact of EPHB2 knockdown on cell proliferation.

### 2.13 Cell scratch experiment

RL95-2 EC cells silencing was seeded into six-well plates, each containing 100,000 cells. Once the cells reached 90% confluency, a scratch was made in the center of each well with a sterile 200 μL pipette tip. After washing with PBS to remove debris, fresh medium was added. Images were taken at 0 and 24 h using a microscope. The scratch width and area were measured with ImageJ software, and the cell migration rate was calculated.

### 2.14 Transwell assay

To assess the invasion ability of RL95-2 EC cells, a Transwell assay was performed using 8 μm pore size inserts (Corning; Catalog No. 3422, USA) coated with Matrigel (Corning; Catalog No. 354234, USA). Cells were transfected with (Dharmacon, USA) and seeded in the upper chamber of the inserts with serum-free RPMI-1640 (Gibco, USA), while the lower chamber contained RPMI-1640 with 10% FBS as a chemoattractant. After 24 h of incubation at 37°C with 5% CO_2_, non-invading cells were removed, and invading cells on the lower surface were fixed with formaldehyde (Sigma-Aldrich, USA), stained with crystal violet (Sigma-Aldrich, USA), and counted under a microscope to quantify cell invasion.

### 2.15 TUNEL assay

To assess apoptosis in transfected RL95-2 EC cells, a TUNEL assay was performed using the *In Situ* Cell Death Detection Kit, Fluorescein (Catalog No. 11684795910; Roche, Switzerland). Cells were transfected (Dharmacon, USA), seeded on glass coverslips, and fixed with 4% paraformaldehyde (Sigma-Aldrich, USA). After permeabilization, cells were stained with the TUNEL reaction mixture and counterstained with DAPI (Sigma-Aldrich, USA). Coverslips were mounted with ProLong™ Gold Antifade Mountant (Thermo Fisher Scientific, USA) and examined under a fluorescence microscope. Apoptotic cells were quantified by counting TUNEL-positive nuclei relative to total DAPI-stained nuclei.

### 2.16 Statistical analysis

All data were processed and visualized using R software (version 4.3.0). Survival analyses were performed using Kaplan–Meier estimation and Cox proportional hazards regression models. Correlation analyses were conducted using Spearman’s rank correlation coefficient. Experimental results are presented as mean ± standard deviation from three independent biological replicates. Statistical significance was determined by either Student’s *t*-test (for two-group comparisons) or two-way ANOVA (for multiple group comparisons), with all calculations performed using GraphPad Prism (version 8.0.2). The following significance levels were applied: ns (not significant), *p < 0.05, **p < 0.01, ***p < 0.001, and ****p < 0.0001.

## 3 Results

### 3.1 EPHB2 expression in EC and its correlation with pathological staging

**Figure 1:**
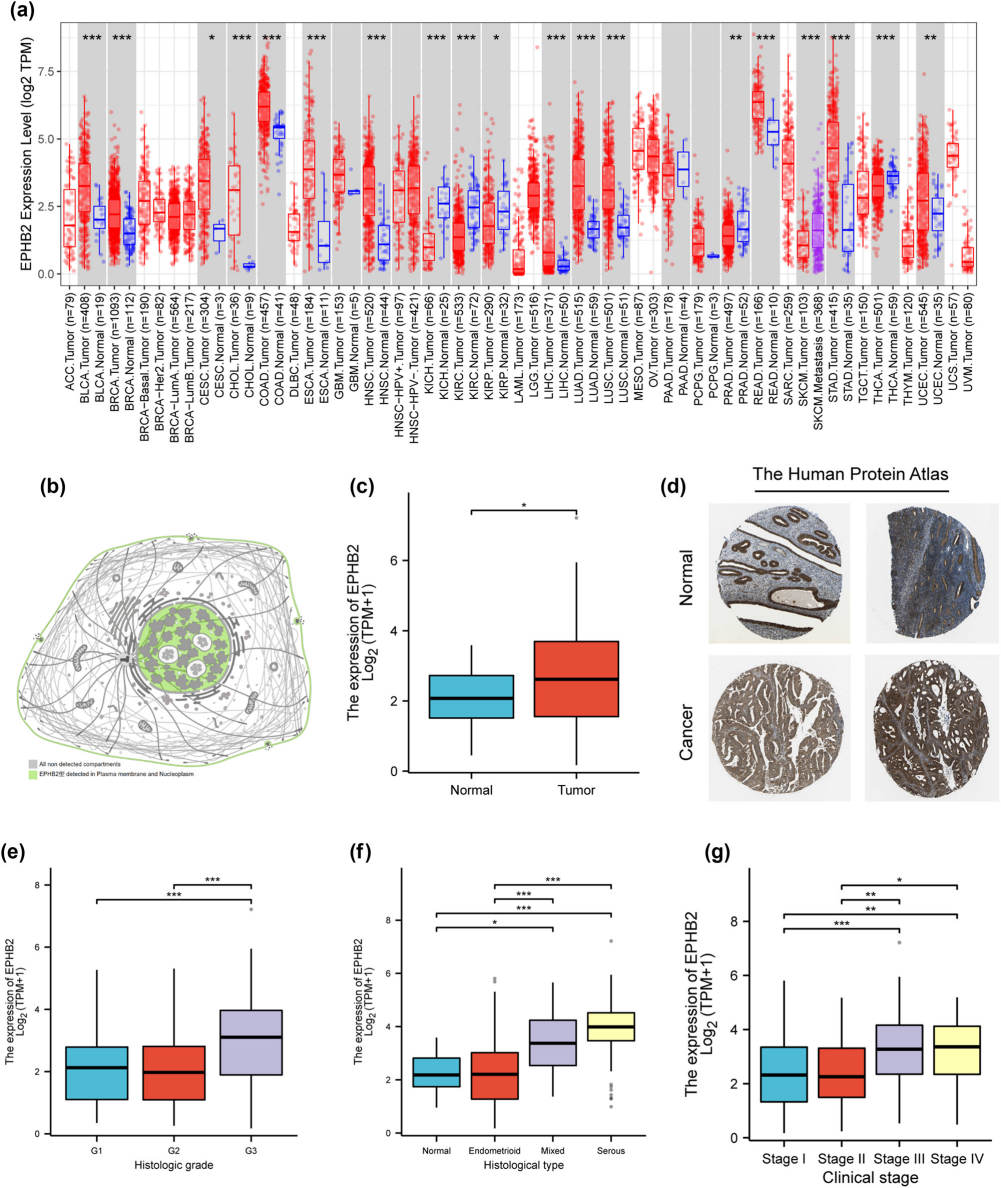
(a) Timer2.0 was used to analyze EPHB2 mRNA expression across diverse cancer types. (b) HPA database was utilized to illustrate EPHB2 subcellular localization in human cells. (c) Box plots depict mRNA expression levels of EPHB2 in EC versus normal endometrium, derived from the TCGA cohort (the total sample size *n* = 595 (including 560 tumor samples and 35 adjacent normal tissue samples). (d) IHC staining images from the HPA database show EPHB2 protein expression patterns in EC tissues. (In normal endometrial tissue, EPHB2 mainly exhibits weak to moderate cytoplasmic staining (brown), and positive cells are sparse. In tumor tissue, however, we observed significantly stronger cytoplasmic and membrane staining (dark brown), and the density of positive cells was significantly higher.) (e)–(g) Box plots further reveal correlations between EPHB2 mRNA expression and EC pathological grade (e), histological type (f), and clinical stage (g), all analyzed using the TCGA cohort. *p < 0.05, **p < 0.01, ***p < 0.001.

In this study, the mRNA expression of EPHB2 across various cancers was analyzed and visualized using Timer2.0 based on data from the TCGA database. Results indicated statistically significant differential expression of EPHB2 mRNA in bladder urothelial carcinoma, breast invasive carcinoma, cervical squamous cell carcinoma and endocervical adenocarcinoma, cholangiocarcinoma, colon adenocarcinoma, esophageal carcinoma, head and neck squamous cell carcinoma, kidney chromophobe, kidney renal clear cell carcinoma, kidney renal papillary cell carcinoma, liver hepatocellular carcinoma, lung adenocarcinoma, lung squamous cell carcinoma, prostate adenocarcinoma, rectum adenocarcinoma, skin cutaneous melanoma, stomach adenocarcinoma, thyroid carcinoma, and uterine corpus endometrial carcinoma (Figure 1a). Additionally, protein expression analysis revealed that EPHB2 is predominantly located in the plasma membrane and nucleus (Figure 1b). Notably, EPHB2 mRNA was significantly overexpressed in endometrial carcinoma (Figure 1c). Immunohistochemical data from the HPA database corroborated these findings, showing higher protein levels of EPHB2 in endometrial carcinoma compared to normal tissues (Figure 1d). Moreover, our analysis revealed a significant correlation between EPHB2 mRNA expression and the pathological grade (Figure 1e), pathological type (Figure 1f), and clinical stage (Figure 1g) of EC.

### 3.2 High expression of EPHB2 in EC is associated with poor prognosis

**Figure 2:**
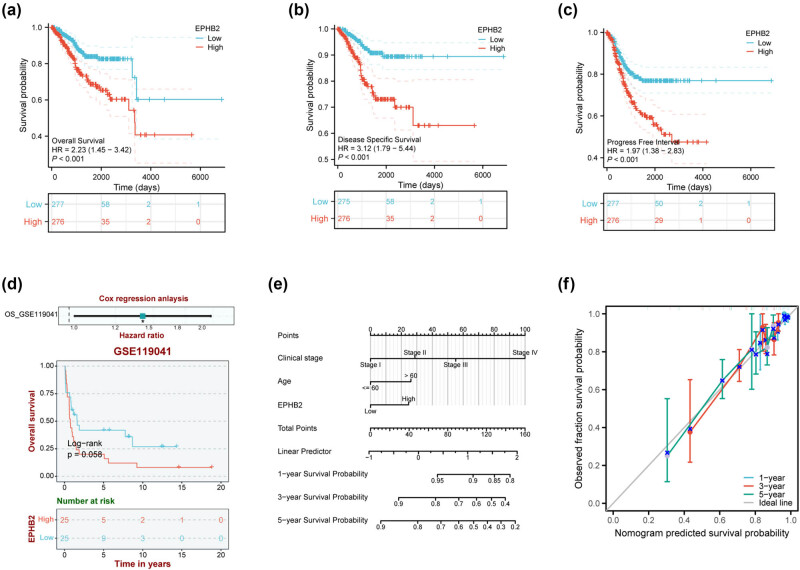
Influence of EPHB2 expression on the survival of patients with (a) OS, (b) DSS, and (c) PFI EC (the total sample size *n* = 595 (including 560 tumor samples and 35 adjacent normal tissue samples). (d) Relationship between EPHB2 expression and OS in GEO (GSE119041) data. (e) A prognostic model for EC was constructed using EPHB2 expression as the Cox factor. (f) Predictive calibration curve used to verify the predictive ability of the model. *p < 0.05.

EPHB2 overexpression demonstrates significant prognostic implications in EC. Through comprehensive survival analysis, we systematically evaluated the association between EPHB2 expression levels and multiple clinical endpoints, including OS, DSS, and PFI. Kaplan–Meier survival curves revealed that patients with high EPHB2 expression exhibited markedly shorter OS (Figure 2a, *p* < 0.001), DSS (Figure 2b, *p* < 0.001), and PFI (Figure 2c, *p* < 0.001) compared to low-expression counterparts.

To validate these findings, we extended our analysis to independent datasets. Using the BEST database, we identified consistent prognostic trends in gene expression omnibus (GEO) dataset GSE119041, where elevated EPHB2 expression similarly predicted worse OS outcomes (Figure 2d, *p* < 0.05).

Furthermore, we developed a robust prognostic model incorporating EPHB2 expression as a key covariate in Cox regression analysis. This model demonstrated excellent predictive performance for 1-, 3-, and 5-year survival rates in EC patients (Figure 2e). The model’s clinical utility was further confirmed through calibration curve analysis, which exhibited strong agreement between predicted and observed survival probabilities (Figure 2f).

### 3.3 EPHB2 interacts with multiple proteins and performs diverse functions in EC

**Figure 3:**
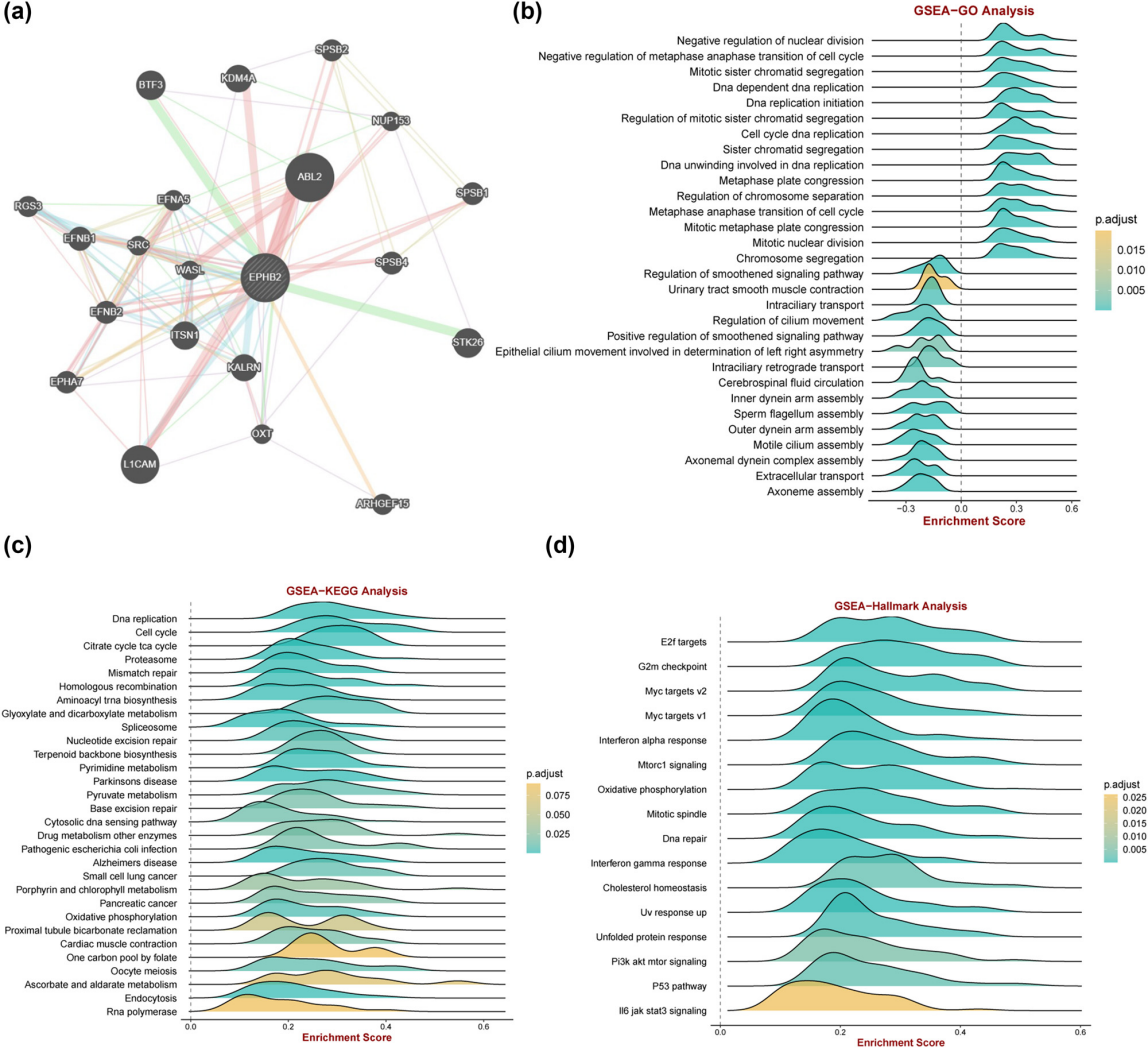
(a) GeneMANIA constructs the interaction network of EPHB2. GO, KEGG, and Hallmark functional enrichment based on GSEA: (b) results of GO enrichment analysis, (c) results of KEGG enrichment analysis, and (d) results of Hallmark enrichment analysis.

To explore the potential functions of EPHB2 in EC, we conducted GeneMANIA analysis, revealing strong interactions between EPHB2 and proteins such as L1CAM, ABL2, KDM4A, BTF3, and SRC, suggesting a functional network (Figure 3a). Further functional exploration using GO, KEGG, and Hallmark enrichment analyses based on GSEA showed significant associations with various biological processes. GO analysis indicated that EPHB2 expression is positively correlated with negative regulation of nuclear division, mid- and late-cell cycle transitions, mitotic sister chromatid segregation, and DNA-dependent DNA replication, while negatively correlated with smoothened signaling pathway regulation, urothelial smooth muscle contraction, intraciliary transport, and ciliary motility (Figure 3b). KEGG analysis revealed significant correlations with DNA replication, cell cycle, citric acid (TCA) cycle, proteasome, and mismatch repair pathways (Figure 3c). Hallmark analysis showed that E2F targets, G2M checkpoint, MYC targets v2-v1, and interferon response were significantly associated with EPHB2 expression in EC (Figure 3d).

### 3.4 EPHB2 mutations and association with immune infiltration in EC

**Figure 4:**
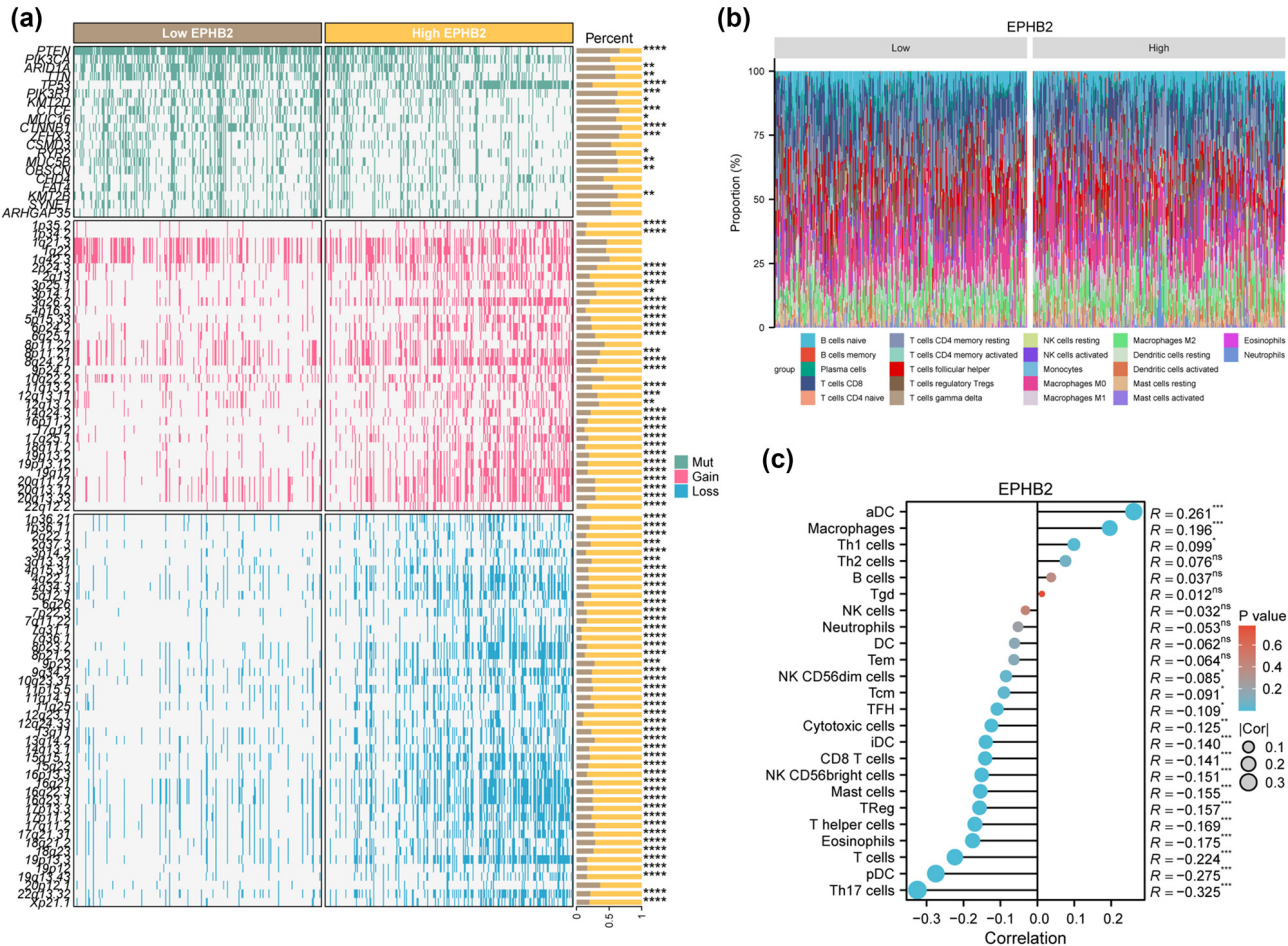
(a) BEST online database assesses genomic mutations in EC patients with high and low expression of EPHB2. (b) Sento Academic online tool assessing immune cell occupancy in EC patients with high and low expression of EPHB2. (c) Lollipop plot showing the correlation of EPHB2 expression with different immune cells. *p < 0.05, **p < 0.01, ***p < 0.001, and ****p < 0.0001.

Genomic mutations and immune cell infiltration are critical factors in tumor progression. In this study, we evaluated the genomic mutations and immune infiltration associated with EPHB2 in EC. Our analysis using the BEST online database revealed significant mutations in genes such as PTEN, PIK3CA, ARID1A, TTN, TP53, and CHD4 in EC patients with high EPHB2 expression, primarily exhibiting gains or losses (Figure 4a). Additionally, we observed significant differences in the immune cell infiltration percentages between patients with high and low EPHB2 expression (Figure 4b). Further analysis demonstrated a significant positive correlation between EPHB2 expression and activated dendritic cells (aDC) (*R* = 0.261) and macrophages (*R* = 0.196). Conversely, there was a significant negative correlation between EPHB2 expression and helper T17 cells (*R* = −0.325), pDC (*R* = −0.275), and T cells (*R* = −0.224) (Figure 4c).

### 3.5 High expression of EPHB2 mRNA and protein in multiple EC cell lines

**Figure 5:**
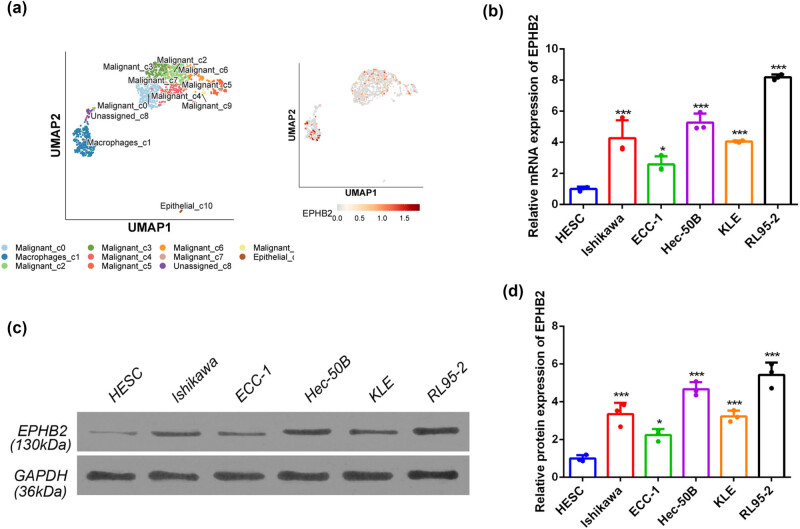
(a) Single cell transcriptome data identifying the mode of expression of EPHB2 in EC. (b) RT-qPCR results, from left to right, HNEC, Ishikawa, ECC-1, Hec-50B, KLE, and RL95-2 (*n* = 3). (c) WB results, from left to right, HNEC, Ishikawa, ECC-1, Hec-50B, KLE, and RL95-2 (*n* = 3). (d) Bar graph showing grey value analysis of WB stripe protein expression (*n* = 3). *p < 0.05, ***p < 0.001.

To determine the significance of EPHB2 in EC, we assessed its mRNA and protein expression across various EC cell lines. Single-cell transcriptomic data analysis initially revealed substantial EPHB2 expression in malignant cells (Figure 5a). We then compared the mRNA and protein expression levels of EPHB2 in EC cell lines (Ishikawa, ECC-1, Hec-50B, KLE, and RL95-2) to those in normal human endometrial stromal cells. qPCR results indicated that EPHB2 mRNA expression was significantly elevated in the EC cell lines relative to normal stromal cells (Figure 5b). Additionally, western blot analysis confirmed that EPHB2 protein levels were markedly higher in the EC cell lines (Figure 5c). Densitometric analysis further demonstrated that the increased EPHB2 protein expression in these cancer cell lines was statistically significant (Figure 5d).

### 3.6 EPHB2 promotes the growth of EC cell line RL95-2

**Figure 6:**
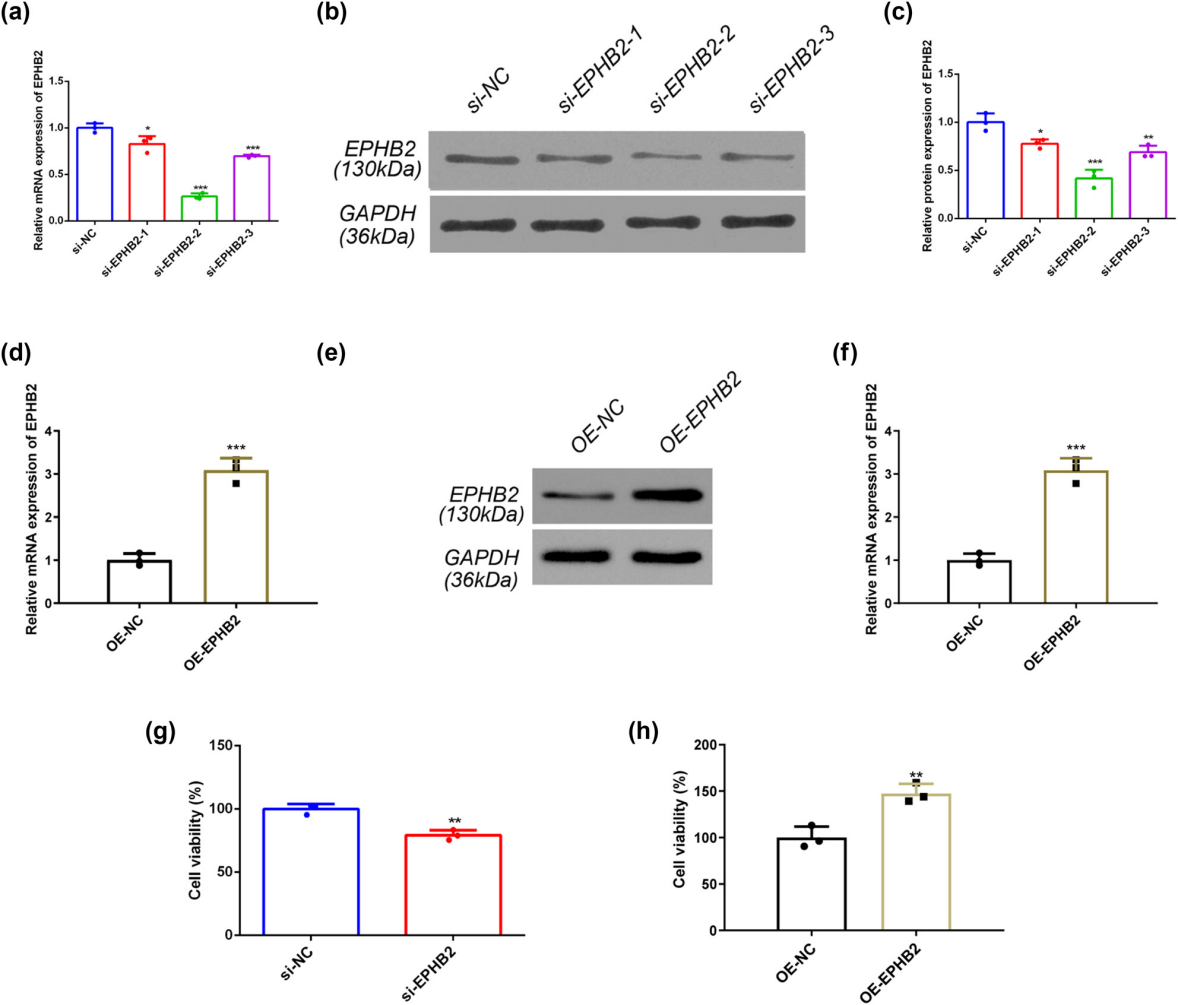
(a) RT-qPCR identified the effects of si-NC, si-EPHB2-1, si-EPHB2-2, and si-EPHB2-3 (*n* = 3). (b) Bar graph showing grey value analysis of WB stripe protein expression (si-NC, si-EPHB2-1, si-EPHB2-2, and si-EPHB2-3) (*n* = 3). (c) WB identification of the effects of si-NC, si-EPHB2-1, si-EPHB2-2, and si-EPHB2-3 (*n* = 3). (d) RT-qPCR identified the effects of OE-NC and OE-EPHB2 (*n* = 3). (e) Bar graph showing grey value analysis of WB stripe protein expression (OE-NC and OE-EPHB2) (*n* = 3). (f) WB identification of the effects of OE-NC and OE-EPHB2 (*n* = 3). (g) and (h) Results of the CCK-8 experiment (*n* = 3). *p < 0.05, **p < 0.01, ***p < 0.001.

To investigate the functional role of EPHB2 in EC, we established stable RL95-2 cell models with modulated EPHB2 expression. Using siRNA-mediated knockdown (si-EPHB2) and overexpression (OE-EPHB2) approaches, we achieved efficient modulation of EPHB2 levels. Quantitative PCR and western blot analyses confirmed significant downregulation of both EPHB2 mRNA (Figure 6a) and protein (Figure 6b and c) expression in si-EPHB2-transfected cells, with si-EPHB2-2 demonstrating the most potent knockdown efficiency. Conversely, OE-EPHB2 transfection resulted in marked upregulation of EPHB2 mRNA (Figure 6d) and protein (Figure 6e and f) expression.

**Figure 7:**
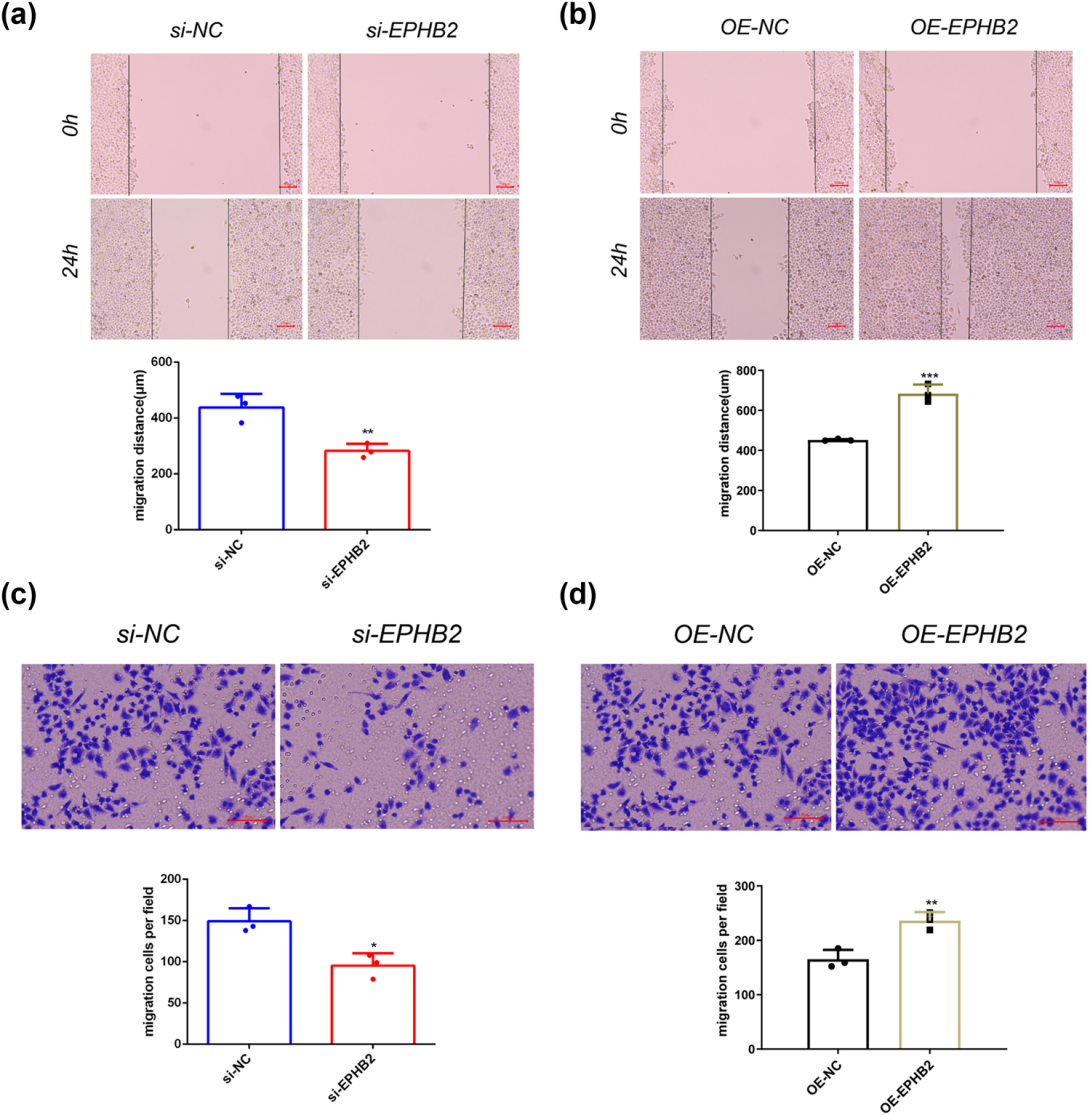
(a) Results of the cell scratch assay after knocking down EPHB2, the bar chart shows the cell migration distance after the scratch (unit: µM; Scale: 100 µM (*n* = 3). (b) Results of the cell scratch experiment after overexpression of EPHB2, the bar graph shows the cell migration distance after the scratch (unit: µM; Scale: 100 µM (*n* = 3). (c) Transwell assay results after knocking down EPHB2 (scale: 100 µM). The bar chart indicates the number of cells invading the compartment (*n* = 3). (d) Transwell assay results after overexpression of EPHB2 (scale: 100 µM). The bar chart indicates the number of cells invading the compartment (*n* = 3). *p < 0.05, **p < 0.01, and ***p < 0.001.

We subsequently performed comprehensive functional assays to characterize the biological consequences of EPHB2 modulation. Cell proliferation assays revealed that si-EPHB2 treatment significantly inhibited RL95-2 cell growth at 24 h post-transfection (Figure 6g), while OE-EPHB2 treatment significantly enhanced proliferation (Figure 6h). Wound healing assays demonstrated that EPHB2 knockdown markedly impaired cell migration capacity (Figure 7a), whereas EPHB2 overexpression significantly promoted migratory potential (Figure 7b).

**Figure 8:**
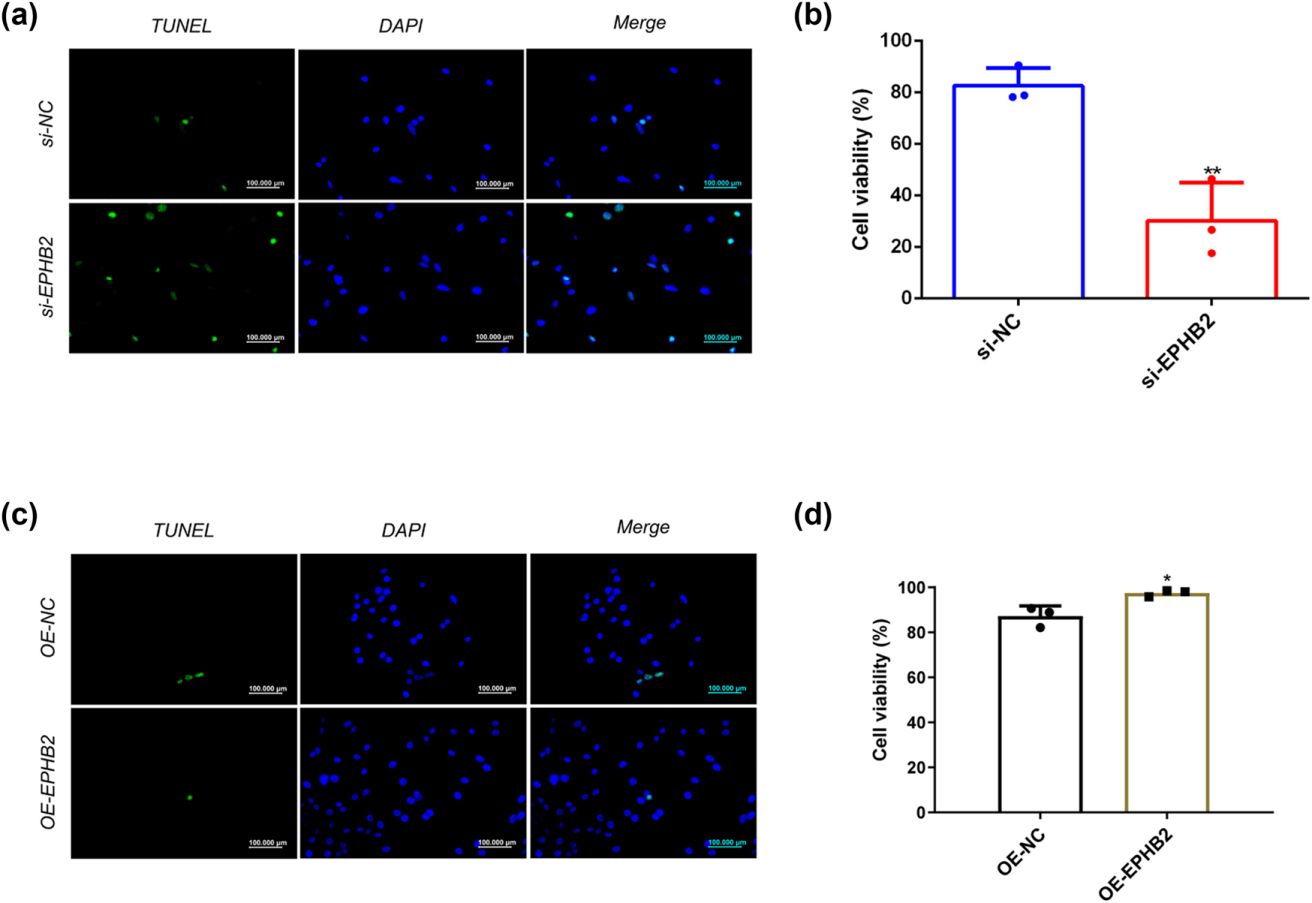
(a) TUNEL experiment results after knocking down EPHB2 (green: TUNAL; blue: DAPI; Scale: 100 µM (*n* = 3). (b) The bar chart shows the percentage of apoptosis. (c) TUNEL experimental results after overexpression of EPHB2 (green: TUNAL; blue: DAPI; Scale: 100 µM. (d) The bar chart shows the percentage of apoptosis (*n* = 3). *p < 0.05, **p < 0.01.

Transwell invasion assays further validated these findings, showing that si-EPHB2 treatment significantly reduced invasive capacity (Figure 7c), while OE-EPHB2 treatment significantly enhanced invasion (Figure 7d). Finally, TUNEL apoptosis assays demonstrated that EPHB2 knockdown significantly increased apoptotic cell death (Figure 8a and b), whereas EPHB2 overexpression significantly decreased apoptosis (Figure 8c and d).

**Figure 9:**
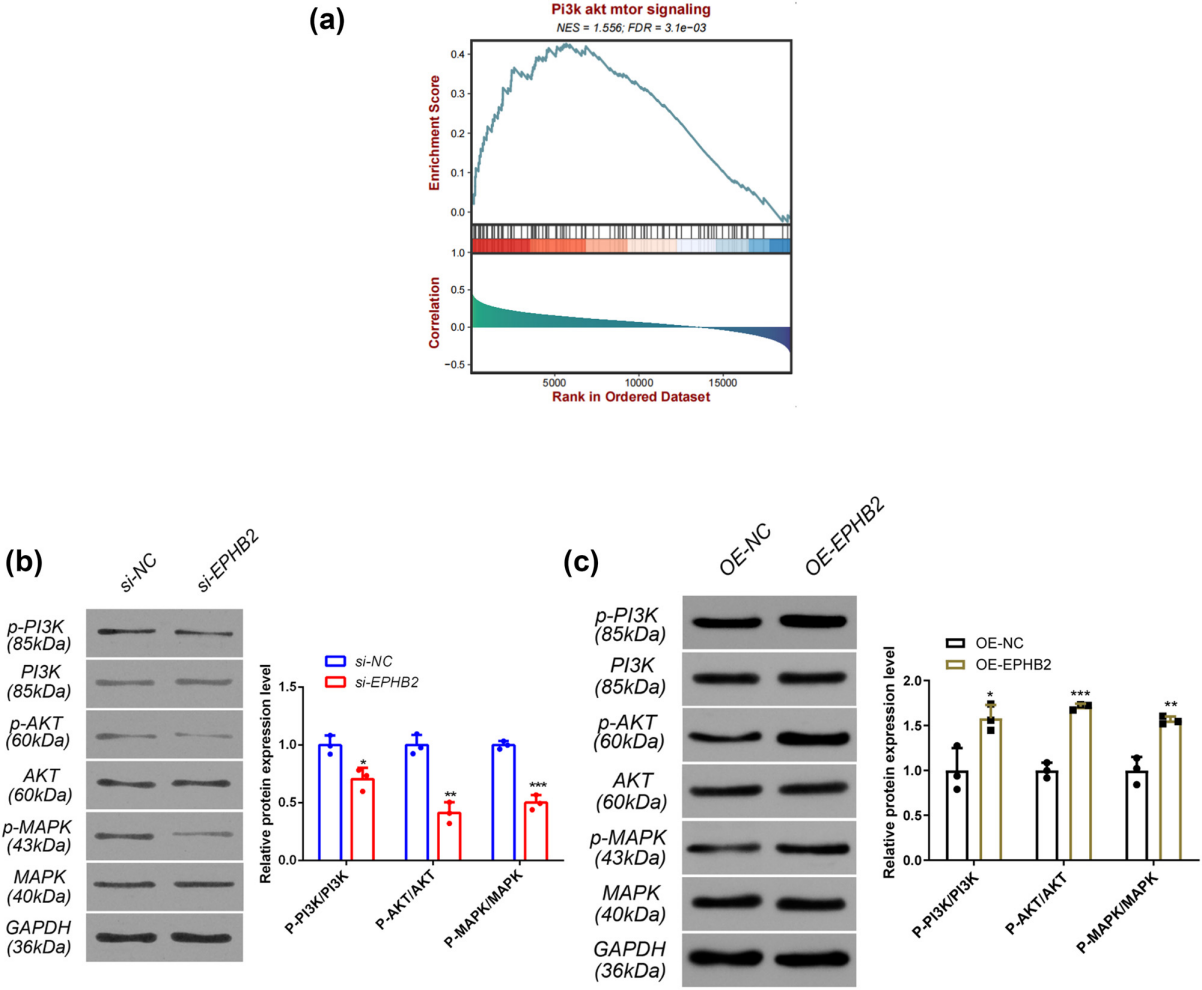
(a) Bioinformatics of GSEA enrichment analysis. (b) After knockdown of EPHB2, GAPDH was used as the internal reference, and the protein expressions of PI3K/AKT/MAPK and p-PI3K/AKT/MAPK were detected by WB. Gray-scale analysis of relative protein expression in the WB band after knocking down EPHB2, and comparison of p-PI3K/PI3K, p-AKT/AKT, and p-MAPK/MAPK. (c) After overexpression of EPHB2, GAPDH was used as the internal reference, and the protein expressions of PI3K/AKT/MAPK and p-PI3K/AKT/MAPK were detected by WB. After overexpression of EPHB2, the relative protein expression in the WB band was analyzed by gray-scale analysis, and p-PI3K/PI3K, p-AKT/AKT, and p-MAPK/MAPK were compared. (*n* = 3), *p < 0.05, **p < 0.01, ***p < 0.001.

To elucidate the mechanism by which si-EPHB2 inhibits the growth of the EC cell line RL95-2, we conducted preliminary mechanistic studies. Bioinformatic GSEA enrichment analysis revealed a significant correlation between EPHB2 and the PI3K/AKT/MAPK signaling pathway (NES = 1.556, FDR = 3.1 × 10^−3^) (Figure 9a). Following transfection of si-EPHB2 into RL95-2 cells, we performed western blot analysis using si-NC as a control. The results showed that the expression of phosphorylated PI3K, AKT, and MAPK proteins was inhibited. Density analysis further confirmed that the reduction in phosphorylated PI3K, AKT, and MAPK proteins was statistically significant compared to total protein levels (Figure 9b). Following OE-EPHB2 in RL95-2 cells, we performed western blot analysis using OE-NC as a control. The results showed that the expression of phosphorylated PI3K, AKT, and MAPK proteins was significantly activated. Density analysis further confirmed that the increase in phosphorylated PI3K, AKT, and MAPK proteins was statistically significant compared to total protein levels (Figure 9c).

## 4 Discussion

Although the rate of early diagnosis of EC is relatively high, a significant number of patients are still diagnosed at an advanced stage, resulting in poor prognosis and treatment outcomes [28]. Biomarkers are crucial in EC research for aiding in prognostic assessment, guiding treatment decisions, and monitoring disease progression and recurrence [29]. Additionally, biomarker studies can uncover key molecular mechanisms and provide a foundation for developing novel therapeutic targets, thereby facilitating the translation of EC research from basic science to clinical application. In this study, we identified that EPHB2 is highly expressed in EC and exerts multiple biological functions, as demonstrated by bioinformatics and experimental validation. Moreover, EPHB2 is associated with poor patient prognosis, and reducing its expression may inhibit tumor progression.

EPHB2 is highly expressed in EC and significantly impacts the pathological stage and patient prognosis. In this study, we observed that both mRNA and protein levels of EPHB2 were elevated in EC tissues compared to normal tissues. Furthermore, our findings demonstrated that EPHB2 expression was significantly correlated with pathological grade, pathological type, and clinical stage of EC. High expression of EPHB2 was associated with poor prognosis, as patients with elevated EPHB2 levels had significantly lower OS, DSS, and PFI. Notably, our prognostic model, which utilized EPHB2 expression as a Cox regressor, effectively predicted the 1-, 3-, and 5-year survival rates of EC patients. This underscores the potential of EPHB2 as a valuable biomarker for prognostic assessment and highlights its role in disease progression. By understanding the molecular mechanisms through which EPHB2 contributes to tumor aggressiveness and poor outcomes, we can develop targeted therapeutic strategies that may improve the prognosis and management of EC. The integration of EPHB2 expression data into clinical practice could enhance personalized treatment approaches, ultimately leading to better patient outcomes.

EPHB2 plays a multifaceted role in EC. We determined that EPHB2 has significant interactions with several key proteins, including L1CAM, ABL2, KDM4A, BTF3, and SRC, suggesting that it may form a functional network critical to cancer progression. Functional enrichment analysis based on GSEA revealed that EPHB2 is associated with various biological processes in EC, including negative regulation of nuclear division, mitotic sister chromatid segregation, regulation of smooth signaling pathway, regulation of cilia movement, DNA replication, cell cycle, TCA cycle, proteasome activity, E2F targets, G2/M checkpoint, and interferon response. Moreover, our results showed a high prevalence of significant mutations in the genes PTEN, PIK3CA, ARID1A, TTN, TP53, and CHD4 in EC patients with elevated EPHB2 expression. This suggests that EPHB2 may interact with these mutations to influence tumor behavior and patient outcomes. Our bioinformatic analysis revealed a striking correlation between EPHB2 expression levels and immune cell infiltration patterns in EC. Notably, high EPHB2 expression was significantly associated with increased infiltration of several immune cell populations, including aDCs, macrophages, Th17 cells, and T cells. These findings suggest a potential immunomodulatory role for EPHB2 in shaping the tumor microenvironment. However, it is important to acknowledge that these results are based solely on computational predictions from existing databases (TIMER/Sento), without direct experimental validation. Future studies should employ multiparameter flow cytometry or single-cell RNA sequencing on patient samples to experimentally confirm these immune cell alterations.

This study provides the first experimental evidence demonstrating that EPHB2 is highly expressed across multiple EC cell lines, including Ishikawa, ECC-1, Hec-50B, KLE, and RL95-2. This widespread overexpression pattern suggests EPHB2 may represent a conserved molecular feature of endometrial tumorigenesis. The single-cell transcriptomic analysis further refined this observation by revealing EPHB2’s specific enrichment in malignant cells, supporting its potential as a cancer cell-intrinsic driver rather than a bystander alteration. Functionally, our experiments using the RL95-2 model system comprehensively characterized the tumor-promoting effects of EPHB2. The observed inhibition of cell proliferation, migration, and invasion following EPHB2 knockdown, coupled with the induction of apoptosis, establishes EPHB2 as a critical regulator of multiple hallmarks of cancer. These findings align with our bioinformatic analyses showing correlation between high EPHB2 expression and aggressive clinical parameters.

The biological significance of these observations extends beyond cell proliferation. The simultaneous impairment of migratory and invasive capabilities upon EPHB2 suppression suggests that this receptor plays a pivotal role in EC metastasis. The pro-apoptotic effect of EPHB2 knockdown further indicates its involvement in evading cell death, another key hallmark of cancer. Together, these data position EPHB2 as a multifunctional oncogene that simultaneously promotes proliferation, metastasis, and survival – three critical processes in tumor progression.

The PI3K/AKT and MAPK signaling pathways play crucial roles in tumor progression by regulating cell proliferation, survival, and apoptosis, thereby promoting tumor formation and development [30,31]. Aberrant activation of these pathways is common in various cancer types, such as PI3K gene mutations, PTEN inactivation [14,32], and BRAF mutations [33], leading to continuous proliferative and anti-apoptotic signaling. Their interactions enhance tumor cell survival and are significant contributors to tumor resistance and recurrence. Targeted therapies against these pathways, including PI3K, AKT, MEK, and ERK inhibitors, have shown effectiveness in clinical trials for certain cancers and hold potential for improving cancer patient outcomes [34,35]. This study conducted a preliminary mechanistic exploration by using si-EPHB2 in the RL95-2 cell line. We observed that the protein expression of phosphorylated PI3K, AKT, and MAPK was suppressed following EPHB2 knockdown. This suggests that EPHB2 may regulate EC cell behavior through the PI3K/AKT/MAPK signaling pathway.

High EPHB2 expression in EC appears to contribute to the aggressive phenotype of these cells by promoting key oncogenic processes such as proliferation and invasion while inhibiting apoptosis. By interfering with EPHB2 expression, it is possible to disrupt these processes, thereby inhibiting tumor growth and progression. Understanding the specific pathways and mechanisms by which EPHB2 influences tumor behavior can lead to the development of targeted therapies aimed at blocking EPHB2 function. This could provide a novel therapeutic approach for treating patients with EC, particularly those with high EPHB2 expression. The integration of EPHB2-targeted strategies into clinical practice has the potential to improve patient outcomes by offering more personalized and effective treatment options.

## 5 Conclusion

In this study, we found that high expression of EPHB2 in EC was associated with a poor prognosis for patients. In addition, EPHB2 is associated with mutations in multiple oncogenes and may promote the progression of malignant tumors through its interaction with the immune microenvironment. In addition, EPHB2 promotes the proliferation, migration and invasion of tumor cells, inhibits tumor cell apoptosis, and leads to the activation of the PI3K/AKT/MAPK signaling pathway. Therefore, EPHB2 may be a potential target for improving the prognosis and treatment of EC.

## References

[1] Makker V, MacKay H, Ray-Coquard I, Levine DA, Westin SN, Aoki D, et al. Endometrial cancer. Nat Rev Dis Primers. 2021;7(1):88. 10.1038/s41572-021-00324-8 PMC9421940 34887451

[2] Braun MM, Overbeek-Wager EA, Grumbo RJ. Diagnosis and management of endometrial cancer. Am Fam Physician. 2016;93(6):468–74. 26977831

[3] Paleari L. New strategies for endometrial cancer detection and management. Int J Mol Sci. 2023;24(7):6462. 10.3390/ijms24076462 PMC10094696 37047434

[4] Guo XM, Roman LD, Klar M, Wright JD, Matsuo K. Malignant peritoneal cytology in endometrial cancer: a contemporary review. Expert Rev Anticancer Ther. 2022;22(9):947–55. 10.1080/14737140.2022.2105208 35862462

[5] Onstad MA, Schmandt RE, Lu KH. Addressing the role of obesity in endometrial cancer risk, prevention, and treatment. J Clin Oncol. 2016;34(35):4225–30. 10.1200/JCO.2016.69.4638 PMC5455320 27903150

[6] Clarke MA, Long BJ, Del Mar Morillo A, Arbyn M, Bakkum-Gamez JN, Wentzensen N. Association of endometrial cancer risk with postmenopausal bleeding in women: a systematic review and meta-analysis. JAMA Intern Med. 2018;178(9):1210–22. 10.1001/jamainternmed.2018.2820 PMC6142981 30083701

[7] Ali AT. Risk factors for endometrial cancer. Ceska Gynekol. 2013;78(5):448–59. 24313431

[8] Contreras NA, Sabadell J, Verdaguer P, Julià C, Fernández-Montolí ME. Fertility-sparing approaches in atypical endometrial hyperplasia and endometrial cancer patients: current evidence and future directions. Int J Mol Sci. 2022;23(5):2531. 10.3390/ijms23052531 PMC8910633 35269674

[9] Marín-Jiménez JA, García-Mulero S, Matías-Guiu X, Piulats JM. Facts and hopes in immunotherapy of endometrial cancer. Clin Cancer Res. 2022;28(22):4849–60. 10.1158/1078-0432.CCR-21-1564 35789264

[10] Yang Y, Wu SF, Bao W. Molecular subtypes of endometrial cancer: implications for adjuvant treatment strategies. Int J Gynaecol Obstet. 2024;164(2):436–59. 10.1002/ijgo.14969 37525501

[11] Moar K, Pant A, Saini V, Maurya PK. Potential biomarkers in endometrial cancer: a narrative review. Biomarkers. 2023;28(4):358–71. 10.1080/1354750X.2023.2179114 36755526

[12] Urick ME, Bell DW. Clinical actionability of molecular targets in endometrial cancer. Nat Rev Cancer. 2019;19(9):510–21. 10.1038/s41568-019-0177-x PMC7446243 31388127

[13] Singh N, Piskorz AM, Bosse T, Jimenez-Linan M, Rous B, Brenton JD, et al. p53 immunohistochemistry is an accurate surrogate for TP53 mutational analysis in endometrial carcinoma biopsies. J Pathol. 2020;250(3):336–45. 10.1002/path.5375 31829441

[14] Álvarez-Garcia V, Tawil Y, Wise HM, Leslie NR. Mechanisms of PTEN loss in cancer: it’s all about diversity. Semin Cancer Biol. 2019;59:66–79. 10.1016/j.semcancer.2019.02.001 30738865

[15] Du Q, Stow EC, LaCoste D, Freeman B, Baddoo M, Shareef AM, et al. A novel role of TRIM28 B box domain in L1 retrotransposition and ORF2p-mediated cDNA synthesis. Nucleic Acids Res. 2023;51(9):4429–50. 10.1093/nar/gkad247 PMC10201437 37070200

[16] Ballester M, Dubernard G, Lécuru F, Heitz D, Mathevet P, Marret H, et al. Detection rate and diagnostic accuracy of sentinel-node biopsy in early stage endometrial cancer: a prospective multicentre study (SENTI-ENDO). Lancet Oncol. 2011;12(5):469–76. 10.1016/S1470-2045(11)70070-5 21489874

[17] Borghi C, Indraccolo U, Scutiero G, Iannone P, Martinello R, Greco P, et al. Biomolecular basis related to inflammation in the pathogenesis of endometrial cancer. Eur Rev Med Pharmacol Sci. 2018;22(19):6294–9. 10.26355/eurrev_201810_16038 30338797

[18] Yu Z, Zhang J, Zhang Q, Wei S, Shi R, Zhao R, et al. Single-cell sequencing reveals the heterogeneity and intratumoral crosstalk in human endometrial cancer. Cell Prolif. 2022;55(6):e13249. 10.1111/cpr.13249 PMC9201371 35560676

[19] Yin FF, Zhao LJ, Ji XY, Duan N, Wang YK, Zhou JY, et al. Intra-tumor heterogeneity for endometrial cancer and its clinical significance. Chin Med J (Engl). 2019;132(13):1550–62. 10.1097/CM9.0000000000000286 PMC6616225 31268882

[20] Liu W, Yu C, Li J, Fang J. The roles of EphB2 in cancer. Front Cell Dev Biol. 2022;10:788587. 10.3389/fcell.2022.788587 PMC8866850 35223830

[21] Jang BG, Kim HS, Chang WY, Bae JM, Kang GH. Prognostic significance of EPHB2 expression in colorectal cancer progression. J Pathol Transl Med. 2018;52(5):298–306. 10.4132/jptm.2018.06.29 PMC6166016 30016858

[22] Zogopoulos G, Jorgensen C, Bacani J, Montpetit A, Lepage P, Ferretti V, et al. Germline EPHB2 receptor variants in familial colorectal cancer. PLoS One. 2008;3(8):e2885. 10.1371/journal.pone.0002885 PMC2483346 18682749

[23] Morales A, Greenberg M, Nardi F, Gil V, Hayward SW, Crawford SE, et al. Loss of ephrin B2 receptor (EPHB2) sets lipid rheostat by regulating proteins DGAT1 and ATGL inducing lipid droplet storage in prostate cancer cells. Lab Invest. 2021;101(7):921–34. 10.1038/s41374-021-00583-9 PMC8217088 33824421

[24] Li T, Fu J, Zeng Z, Cohen D, Li J, Chen Q, et al. TIMER2.0 for analysis of tumor-infiltrating immune cells. Nucleic Acids Res. 2020;48(W1):W509–14. 10.1093/nar/gkaa407 PMC7319575 32442275

[25] Warde-Farley D, Donaldson SL, Comes O, Zuberi K, Badrawi R, Chao P, et al. The GeneMANIA prediction server: biological network integration for gene prioritization and predicting gene function. Nucleic Acids Res. 2010;38(suppl_2):W214–20. 10.1093/nar/gkq537 PMC2896186 20576703

[26] Liu Z, Liu L, Weng S, Xu H, Xing Z, Ren Y, et al. BEST: a web application for comprehensive biomarker exploration on large-scale data in solid tumors. J Big Data. 2023;10(1):165.

[27] Deng Y, Chen P, Xiao J, Li M, Shen J, Qin S, et al. SCAR: Single-cell and spatially-resolved cancer resources. Nucleic Acids Res. 2024;52(D1):D1407–17. 10.1093/nar/gkad753 PMC10767865 37739405

[28] Francoeur AA, Fontenont V, Tewari KS. Treatment options in the advanced and recurrent setting for endometrial cancer: an update. Expert Rev Anticancer Ther. 2024;24(8):731–44. 10.1080/14737140.2024.2370377 38913791

[29] Galant N, Krawczyk P, Monist M, Obara A, Gajek Ł, Grenda A, et al. Molecular classification of endometrial cancer and its impact on therapy selection. Int J Mol Sci. 2024;25(11):5893. 10.3390/ijms25115893 PMC11172295 38892080

[30] He Y, Sun MM, Zhang GG, Yang J, Chen KS, Xu WW, et al. Targeting PI3K/Akt signal transduction for cancer therapy. Signal Transduct Target Ther. 2021;6(1):425. 10.1038/s41392-021-00828-5 PMC8677728 34916492

[31] Fresno Vara JA, Casado E, de Castro J, Cejas P, Belda-Iniesta C, González-Barón M. PI3K/Akt signalling pathway and cancer. Cancer Treat Rev. 2004;30(2):193–204. 10.1016/j.ctrv.2003.07.007 15023437

[32] Carnero A, Blanco-Aparicio C, Renner O, Link W, Leal JF. The PTEN/PI3K/AKT signalling pathway in cancer, therapeutic implications. Curr Cancer Drug Targets. 2008;8(3):187–98. 10.2174/156800908784293659 18473732

[33] Poulikakos PI, Sullivan RJ, Yaeger R. Molecular pathways and mechanisms of BRAF in cancer therapy. Clin Cancer Res. 2022;28(21):4618–28. 10.1158/1078-0432.CCR-21-2138 PMC9616966 35486097

[34] Li Q, Li Z, Luo T, Shi H. Targeting the PI3K/AKT/mTOR and RAF/MEK/ERK pathways for cancer therapy. Mol Biomed. 2022;3(1):47. 10.1186/s43556-022-00110-2 PMC9768098 36539659

[35] McCubrey JA, Steelman LS, Chappell WH, Abrams SL, Wong EW, Chang F, et al. Roles of the Raf/MEK/ERK pathway in cell growth, malignant transformation and drug resistance. Biochim Biophys Acta. 2007;1773(8):1263–84. 10.1016/j.bbamcr.2006.10.001 PMC2696318 17126425

